# Impact of feed glyphosate residues on broiler breeder egg production and egg hatchability

**DOI:** 10.1038/s41598-021-98962-1

**Published:** 2021-09-29

**Authors:** Leslie Foldager, Jeanet F. M. Winters, Natalja P. Nørskov, Martin T. Sørensen

**Affiliations:** 1 Department of Animal Science, Aarhus University, Blichers Allé 20, 8830 Tjele, Denmark; 2 Bioinformatics Research Centre, Aarhus University, C.F. Møllers Allé 8, 8000 Aarhus, Denmark

**Keywords:** Developmental biology, Ecology

## Abstract

Glyphosate is the active substance in glyphosate-based herbicides, e.g. Roundup. Its widespread application on feed crops leaves residues in the feed. Glyphosate has antimicrobial and mineral chelating properties and we investigated whether there is an association between feed residues of glyphosate on the one side and broiler breeder egg laying percent and egg hatchability on the other side. Twenty-six feed samples from five conventional flocks producing hatching eggs were analysed for glyphosate. Data on laying percent and egg hatchability from periods following each feed sampling were then associated with feed residues of glyphosate. The average glyphosate residue level was 0.09 mg/kg, maximum was 0.19 and minimum was 0.004 mg/kg. Average laying percent over observation days was 65% (SD = 5.4%) and average hatchability was 79% (SD = 5.8%). We found a negative association between feed glyphosate residue level and hatchability (*P* = 0.03) when adjusted for breeder age, storage time of eggs on farm before delivery and storage time at hatchery before incubation start. No association was found with laying percent (*P* = 0.59) adjusted for breeder age. The range of glyphosate concentrations in feed was narrow and should be kept in mind when interpreting both significant and non-significant associations with glyphosate residue concentrations. In nine of 24 analysed conventional eggs the concentration of glyphosate in yolk was above the detection limit however below the quantification limit indicating that traces of glyphosate are common in conventional eggs.

## Introduction

Glyphosate is the active substance in glyphosate-based herbicides, e.g. Roundup. Among its applications, glyphosate-based herbicides are used on genetically modified crop and for pre-harvest desiccation of crop. This application leaves residues in feed for livestock. Since glyphosate is known to inhibit gut bacteria to various degree in vitro^1^ and to have mineral chelating properties^2^, it was speculated that glyphosate residues in feed might affect the gut microbiome and mineral availability, potentially in an unfavourable direction for egg production and fertility. On this background, we investigated whether there is an association between feed residues of glyphosate on the one side and egg laying percent (i.e. the percentage of breeders laying egg) and hatchability (defined as the proportion of viable chicken hatched from eggs placed in incubators) on the other side. Indeed Ruuskanen et al.^3^ recently indicated an adverse effect of the glyphosate-based herbicide Roundup on embryo development in birds, i.e. an effect that likely would influence egg hatchability.

Two outcomes were investigated for association with feed residues of glyphosate: (1) laying percent per production day and (2) the hatchability per delivery day calculated as the proportion of viable chickens from eggs placed in incubators. Concentration of glyphosate residue in the feed consumed by the breeders during the 10 days prior to laying was the explanatory variable of main interest. In addition, conventional eggs acquired from grocery stores were analysed for glyphosate residue level.

## Materials and methods

This is an observational study with no intervention on flock and hatchery practices. None of the birds or eggs were exposed to experimental procedures. The study was based mainly on existing data provided by the hatchery company (DanHatch Denmark A/S) from five broiler breeder flocks in Denmark during the period from November 2018 to January 2019 when the breeders were 46 to 62 weeks of age, see details in Table 1. In addition, feed samples from the flock locations and eggs from grocery stores were acquired.

**Table 1:** Flocks and production periods.

Flock (ID)	Feed samples (N)	Egg laying period^a^ (first-last date)	Number of breeders (min–max; mean; SD)	Breeder age in weeks (min–max; mean; SD)
1	10	19 Nov 2018–23 Dec 2018	39,026–39,325; 39,166; 87.3	45.6–50.4; 48.0; 1.46
2	5	6 Dec 2018–7 Jan 2019	30,878–31,288; 31,033; 121.8	57.0–61.6; 59.3; 1.38
3	3	2 Dec 2018–13 Dec 2018	31,478–31,633; 31,550; 55.3	58.6–60.1; 59.4; 0.52
4	5	14 Dec 2018–24 Jan 2019	32,551–32,741; 32,647; 54.9	48.1–54.0; 51.1; 1.75
5	3	7 Dec 2018–29 Jan 2019	20,569–20,721; 20,647; 46.6	47.1–54.7; 50.9; 2.25

^a^First feed sample was obtained 10 days before the first day of the period.

The average age of breeders was 48–59 weeks (SD from 0.5 to 2.2) ranging from 46–50 weeks to 57–62 weeks (Table 1; Supplementary Fig. S1 online) with observation period ranging from 1.6 to 7.6 weeks in the five flocks. Average laying percent over observation days was 65% (SD = 5.4%) and average hatchability over deliveries was 79% (SD = 5.8%).

### Feed samples

Twenty-six feed samples were collected for analysis of glyphosate content, 3 to 10 feed samples per flock. The glyphosate concentration related to a given sampling date was assumed representative for the flock from this day and until next sampling. Average duration of the preceding samples were used as duration for the last sampling date within each flock. Glyphosate (*N*‐(phosphonomethyl) glycine) and the glyphosate degradation product, aminomethylphosphonic acid (AMPA) in feed samples were analysed by the method described by Nørskov et al.^4^.

### Production data

Data on egg production and hatchability from periods following each feed sampling was obtained from the hatchery company. Daily information was available on laying percent (100% * number of eggs/number of breeders), breeder age (days) and egg weight. For the hatchability, this was calculated as the proportion of eggs placed in incubators from which a viable chicken hatched (but presented as a percentage, i.e. multiplied by 100%). Daily egg weight had been calculated as the average from approx. 30 randomly sampled eggs.

Glyphosate concentration of the feed consumed by the breeders during the 10 days prior to laying was the explanatory variable of main interest. The weighted average of glyphosate concentrations across the 10 days of development from follicle to ovulation of egg was used with number of days each glyphosate sample is representative during these 10 days as weights. For hatchability, glyphosate concentrations were aggregated at the level of delivery by weighted averaging using number of hatch eggs as weights.

### Eggs from grocery stores

No eggs were obtained from the five flocks, however we acquired eight cartons of conventional as well as eight cartons of organic eggs from eight different grocery stores. Three eggs from each carton were selected and egg yolk were analysed for glyphosate by the microLC-MS/MS method as described by Nørskov et al.^4^ adjusted to the egg yolk matrix.

### Statistical analysis

Laying percent and hatchability were analysed by linear mixed effects models, including a random effect of flock and a first order autoregressive correlation structure to account for the repeated measurements from each flock. Following two covariates were considered for both outcomes: average egg weight (g) and breeder age (decimal weeks). However, since egg weight and breeder age are highly correlated (Pearson’s correlation coefficient ranging from 0.73 to 0.95 in the five flocks; Supplementary Fig. S1 online), only breeder age was included in the models. An important reason for this choice being that average egg weight was missing for 24% and 43% of the days from flock 4 and 5, respectively. In the age range used for this study, laying percent decrease with breeder age (Supplementary Fig. S1 online) as substantiated by a correlation coefficient between − 0.38 and − 0.87. Hatchability also decrease with breeder age (Supplementary Fig. S1 online).

In addition, storage time on farm until delivery (1 to 5 days) and storage time at hatchery until incubation starts (1 to 11 days) were included as covariates for hatchability. The incubation start date was determined as date of hatching minus 21 days. For hatchability, covariates obtained from flock production data were aggregated at the level of delivery by weighted averaging; using daily number of eggs as weights for the calculation of average egg weight, number of hatch eggs as weights for average storage time on farm, and current number of breeders as weights for average breeder age. Weighted average storage time on farm until delivery varied from 1.0 to 4.0 and was on average 2.1 days. For storage time at hatchery, deliveries had been split on one to four incubator start dates. Therefore, weighted average of storage days was calculated using number of delivered eggs as weights. Weighted average storage time at hatchery before incubation starts varied from 1.2 to 8.0 days and was on average 4.8 days.

Final models were fitted with restricted maximum likelihood estimation using the *lme* function from the *nlme* package v. 3.1-152 in R version 4.0.4^5^ and with a significance level of 0.05. Fixed effects were tested by χ^2^ likelihood ratio tests after maximum likelihood estimation. Model checking was carried out by examination of qq-plots for normality and scatter plots of residuals versus predicted values to look for uncovered trends and variance heterogeneity.

## Results and discussion

The average glyphosate residue level of all 26 feed samples was 0.09 mg/kg, maximum was 0.19 and minimum was 0.004 mg/kg. The corresponding statistics for AMPA were 0.05, 0.14 and 0.001 mg/kg. There was a positive and relatively high correlation between glyphosate and AMPA residues with a Pearson’s correlation coefficient of 0.80. Therefore, AMPA was not analysed further. Distribution of glyphosate levels within flock is shown in Supplementary Fig. S1 online.

In nine of the 24 analysed conventional eggs and two of the 24 analysed organic eggs, the concentration of glyphosate in yolk was above the detection limit however below the quantification limit of the applied method (< 0.1 ng/g; Nørskov, N.P., personal communication). These data indicate that traces of glyphosate are common in conventional eggs and cannot be excluded in organic eggs.

**Figure 1:**
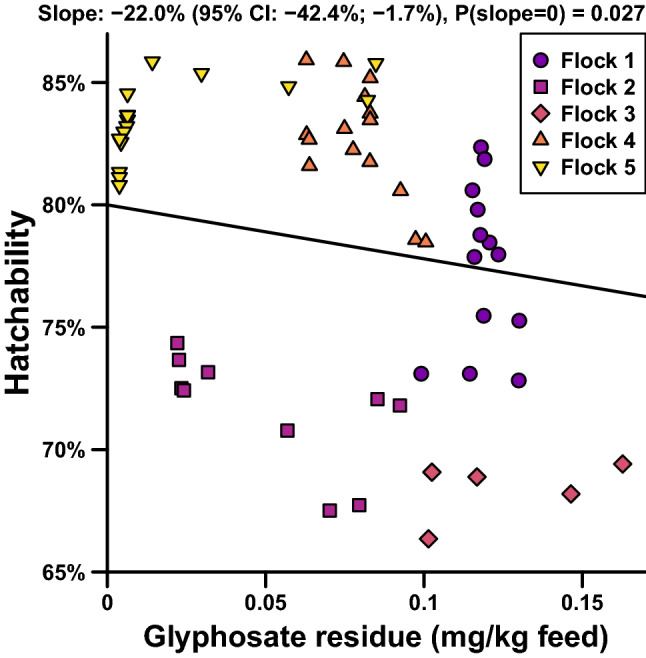
Linear mixed effects model of hatchability. Hatchability as a function of feed residues of glyphosate with adjustment for breeder age, storage times on farm until delivery and storage times at hatchery until incubation starts.

Within the range of feed residue glyphosate investigated, we found a negative association with hatchability (*P* = 0.03) adjusted for breeder age, storage time on farm before delivery and storage time at hatchery before incubation start, see Fig. 1. No association was found with laying percent (*P* = 0.59) adjusted for breeder age. Thus, it seems that feed glyphosate residues adversely affect the fertility of eggs although breeder productivity was not affected. Assuming that the ingredients in diets for laying hens and broiler breeders are basically the same and thus that the levels of glyphosate in eggs from the five included flocks are comparable to the levels in the acquired conventional eggs from grocery stores, even levels not quantifiable by the applied method seem to affect hatchability adversely. Ruuskanen et al.^3^ found a tendency that 200 mg glyphosate per kg feed adversely affected embryo development in a bird model, thus and despite the very different feed levels of glyphosate in that and our investigation, the reduced hatchability might be due to adversely affected embryo development.

It would have been desirable with a wider range of glyphosate residues but this is difficult to guarantee in a commercial setting. Thus, an experiment with glyphosate residues in feed over a range covering the conditions in commercial settings and including feed free of residues is warranted to further investigate potential effects of feed glyphosate residues on egg hatchability and foetal development.

## Conclusion

In conclusion, there was a statistically significant negative association between residues of glyphosate in feed for broiler breeders and egg hatchability while no association was found between residues of glyphosate in the feed and laying percent. The range of glyphosate concentrations in feed was narrow and should be kept in mind when interpreting both significant and non-significant associations with glyphosate concentration. Traces of glyphosate was found to be common in conventional eggs acquired from grocery stores.

## Supplementary Information

Supplementary Information.

## Data Availability

The production data that are a main basis for the findings of this study were provided by the commercial company DanHatch Denmark A/S and so are not publicly available. Data are however available in anonymised form from the authors upon reasonable request and conditional on permission from DanHatch Denmark A/S.
