# Supplementary material for: Impact of feed glyphosate residues on broiler breeder egg production and egg hatchability
Source: Sci Rep. 2021 Sep 29;11:19290. doi: 10.1038/s41598-021-98962-1 (PMC8481226; doi:10.1038/s41598-021-98962-1)
Supplement: Supplementary file 1 — Supplementary Information. [file 41598_2021_98962_MOESM1_ESM.pdf]

Supplementary information

Impact of feed glyphosate residues on broiler breeder egg production and egg hatchability

Leslie Foldager[1,2,*], Jeanet F. M. Winters[1], Natalja P. Nørskov[1], and Martin T. Sørensen[1,*]

[1]Department of Animal Science, Aarhus University, Blichers Allé 20, DK8830 Tjele, Denmark
[2]Bioinformatics Research Centre, Aarhus University, C.F. Møllers Allé 8, DK8000 Aarhus, Denmark
[*]Corresponding authors: leslie@anis.au.dk (L. Foldager; regarding data and statistics), martint.sorensen@anis.au.dk (M.T. Sørensen; regarding biology)

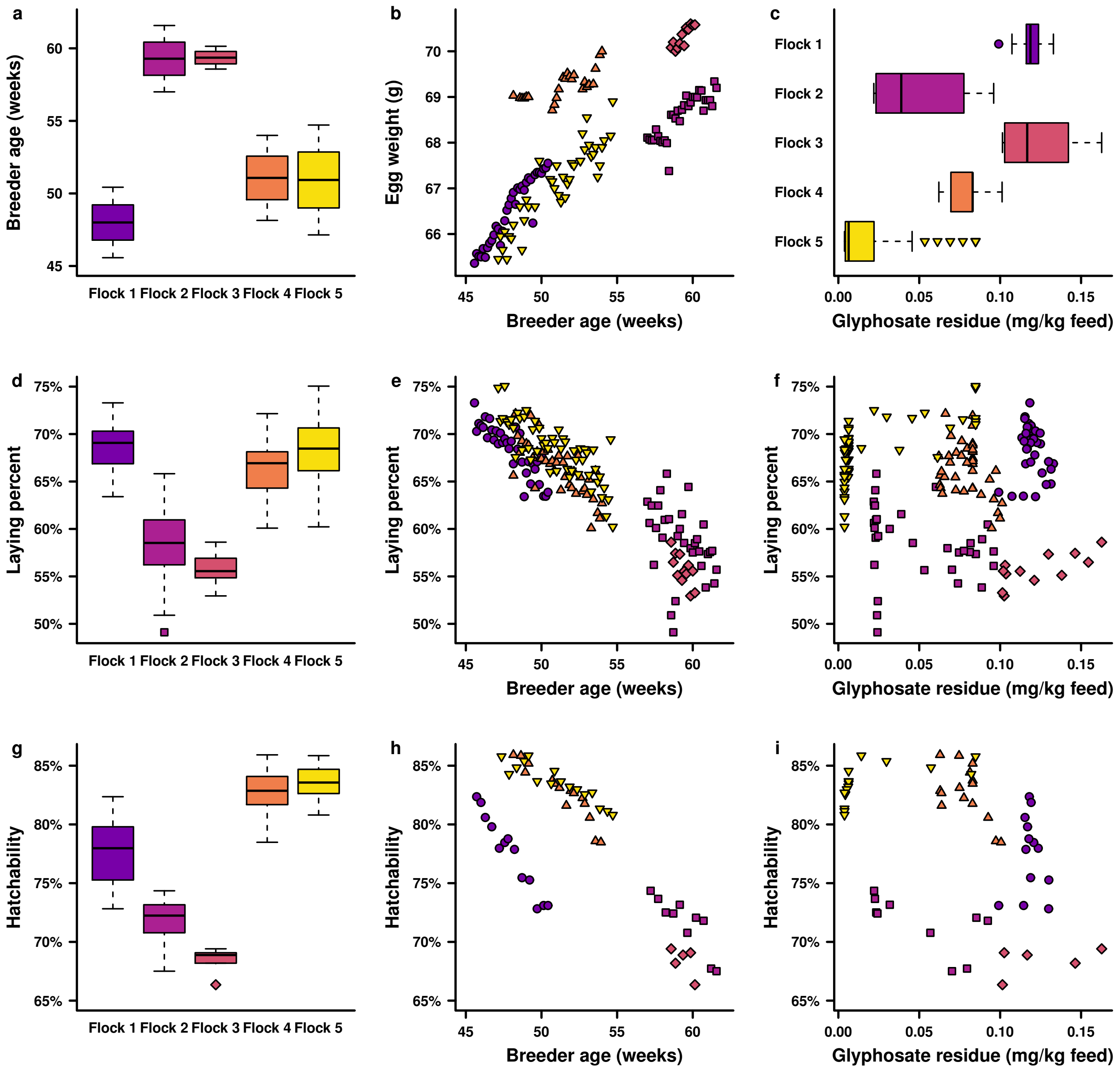

Supplementary Figure S1. Production and glyphosate residue. Box–and–whiskers plots of (**a**) breeder age, (**d**) laying percent, (**g**) hatchability and (**c**) glyphosate residue in feed for each flock. The lower and upper edges of a box ('hinges') are versions of first and third quartile, the line inside the box is the median and the broken lines extending from the box (whiskers) are the most extreme point which is no more than 1.5 times the length of the box away from the box. Points outside the whiskers (outliers) are also plotted. Scatter plots showing relation between breeder age and respectively (**b**) egg weight, (**e**) laying percent and (**h**) hatchability, and between glyphosate residue in feed and respectively (**f**) laying percent and (**i**) hatchability.
